# Serum Extracellular Vesicles Cargo Approach in Bitches with Mammary Tumors

**DOI:** 10.3390/cimb46070459

**Published:** 2024-07-22

**Authors:** Gabriela C. Sousa, Marcos G. Carvalho, Carlos E. Fonseca-Alves, Fabiana F. Souza

**Affiliations:** 1 Department of Veterinary Surgery and Animal Reproduction, School of Veterinary Medicine and Animal Science, São Paulo State University, Unesp, Botucatu 18618-687, São Paulo, Brazil; gc.sousa@unesp.br (G.C.S.); marcos.gomides@unesp.br (M.G.C.); carlos.e.alves@unesp.br (C.E.F.-A.); 2 Department of Small Animal Clinical Sciences, Virginia Maryland College of Veterinary Medicine, Blacksburg, VA 24061, USA

**Keywords:** oncology, protein, exosome, mammary tumor, liquid biopsy

## Abstract

This study investigated serum extracellular vesicles (EVs) in bitches with mammary neoplasms, in order to understand their size, shape, and concentration, as well as their association with tumor malignancy. Thirty bitches were categorized into control (*n* = 10), mammary tumor grades I and II (GI, *n* = 13), and grade III (GII, *n* = 7). Serum was separated from blood collected during mastectomy, and EVs were isolated using size exclusion chromatography. The analysis revealed no significant differences in EV concentrations among groups, with similar concentrations for control, GI, and GII. Ninety-one proteins were identified in EV-enriched samples, with six showing varied abundance across groups. Notably, keratin 18 was highly abundant in GI, while sushi domain-containing protein, EvC ciliary subunit 2, and the joining chain of multimeric IgM and IgA were increased in GII. Additionally, protocadherin 17 and albumin were upregulated in both GI and GII. ROC curves identified potential biomarkers for differentiating tumor grades. Enrichment pathway analysis revealed AFP gene upregulation in the GI. Mass spectrometry proteomics data were deposited in Mendeley Data. The study provides valuable insights into serum EV characterization in bitches, suggesting keratin 18 and protocadherin 17 as potential biomarkers for canine mammary neoplasia, with implications for future diagnostic and therapeutic strategies.

## 1. Introduction

Mammary neoplasia is the most common type of cancer in female dogs and is one of the leading causes of death in this species [1,2,3]. This type of cancer demonstrates malignancy in between 53 and 60% of cases, and in Brazil, the data can reach 67 and 89% [1,4,5]. A similar scenario is observed in women, in 2020 approximately 2.3 million women were diagnosed and 685,000 died from breast cancer worldwide, according to the World Health Organization (WHO). Given this high occurrence, studies that seek to identify biomarkers related to mammary neoplasms are increasing.

In humans, recent studies have identified biomarkers contained in extracellular vesicles (EVs), nanoparticles with a double lipid membrane and spherical shape, which can assist in the early diagnosis, staging, prognosis, and monitoring of different types of cancer since they can reflect the condition of the cell of origin [6,7]. In addition to containing proteins, lipids, and nucleic acids produced by the progenitor cell, EVs can also be isolated from tissues and body fluids, such as plasma and blood serum, saliva, urine, and milk [8,9,10].

The use of EVs as biomarkers has the advantage of higher stability due to protection by the bilipid membrane, preventing degradation of their content by lipases and proteases, and despite being a nanoparticle, the high amount of EVs in body fluids suggests a higher concentration of biomarkers, compared to free biomarkers in body fluids, thus supporting the monitoring of diseases [8,10,11].

EVs have critical roles in metabolic processes, intercellular communication, tissue repair, regulation, and suppression of the immune response, and can assist in elucidating several physiological processes [12,13]. In oncology, EVs have already demonstrated their role in carcinogenesis, promoting intercellular communication and expression of elements involved in tumor growth and proliferation, such as TGF-β, VEGF, and SDF-1. In addition, they are involved in the tumor microenvironment, immunomodulation, and transport of metastatic information [13,14,15].

The stability and abundance of EV cargo have stimulated studies to identify biomarkers that could be used in clinical routine settings in the future. The protein profile of EVs obtained from neoplastic tissues in women was correlated with the subtype of breast neoplasms, a significant advance for diagnosis using non-invasive methods [16]. The protein profile of EVs from blood and plasma of women with breast cancer suggested, as potential biomarkers, serpin 1, KRT6B, and SOCS3 proteins in a favorable and IGF2R in an unfavorable prognosis [17].

In veterinary medicine, EV research has been advancing in the last few years, presenting a vast and promising area to be investigated. In cells from one of the main types of cancer in dogs, osteosarcoma, proteins associated with immunomodulation, such as α-feto protein, TGFβ, HSP90, and HSP70, were upregulated in neoplastic lineages when compared to non-neoplastic lineages, and TGF-β was found only in exosomes from neoplastic lineages [18]. An in vivo study of dogs with osteosarcoma identified more than 170 proteins in serum EVs, correlated with proteolysis, immune system regulation, stress response activation, and regulation of metabolic processes [19].

The concentration of circulating EVs in biofluids has also been investigated, since this could provide significant results using a minimally invasive method. In the blood plasma of dogs with different types of neoplasm, a higher concentration of EVs was found compared to healthy animals [20], as well as in dogs with lymphoma and adenocarcinoma compared to healthy animals. In addition, the dogs with lymphoma had smaller EVs than healthy animals [21].

In mammary tumors, there are still few studies with EVs; however, these nanoparticles derived from mammary gland neoplasm lineages have already been isolated and characterized in dogs and cats [22]. In addition, a study evaluated their autocrine functions and compared different isolation methods [23].

In view of the above and considering the research field to be explored with mammary neoplasms and EVs, the current study aimed, for the first time, to isolate, characterize, and compare the protein profile of EVs in the blood serum of bitches with mammary tumors.

## 2. Materials and Methods

### 2.1. Reagents

All reagents used were of high purity and obtained from Sigma-Aldrich (St. Louis, MO, USA), GE Healthcare Life Science (Sao Paulo, São Paulo, Brazil), Waters Corp. (Barueri, São Paulo, Brazil), and Thermo Fisher Scientific (São Paulo, São Paulo, Brazil), when not cited.

### 2.2. Selection of Animals and Collection of Samples

Thirty bitches were included in the study, ten selected for the control group, according to the inclusion criteria; young females (up to 4 years old), healthy, with no previous history of neoplasms, and who underwent elective ovariohysterectomy. Twenty dogs were selected for the neoplasm groups, according to the inclusion criteria; females aged over 4 years, of all breeds, that had one or multiple mammary nodules, intact or not, and without a previous history of other types of cancer or concomitant diseases. The bitches in the neoplasm groups were previously screened by fine needle aspiration cytology to identify the primary origin of the neoplasm and subsequently underwent unilateral mastectomy surgery in the Animal Reproduction Service of the Veterinary Hospital of the School of Veterinary Medicine and Animal Science, São Paulo State University, São Paulo, Brazil. After surgery and the histological classification of the neoplasm, the group was divided into groups I and II.

As part of the protocol of the Animal Reproduction Sector, the animals that had a mastectomy underwent chest radiography to search for metastasis and an electrocardiogram. In addition, blood was collected from all animals in the control group and groups with neoplasia to perform a complete blood count and renal and hepatic biochemical profiles. All owners answered a questionnaire containing information about the general condition of the animal, time of evolution of breast nodules, reproductive status, history of reproductive diseases or not, and administration of contraceptives and other medications, in addition to signing a term of consent and clarification for inclusion of the animal in the study. Blood for EV separation was collected from the jugular vein during the transoperative period of mastectomy or ovariohysterectomy procedures.

### 2.3. Histopathology, Classification of Tumors, and Division of Groups

All surgically excised mammary chains were sent to the Veterinary Pathology service of the Department of Veterinary Clinic of the Faculty of Veterinary Medicine and Animal Science of São Paulo State University, Campus Botucatu, Botucatu, São Paulo. Breast nodules were subjected to histological analysis and classified according to the characteristics described by Cassali et al. [24,25] and Goldschmidt et al. [26]. In animals with more than one nodule in the same mammary chain, the more severe neoplasm was always considered for the division of neoplasm groups.

The bitches were then separated into 3 groups: (GI) with grade I and II tumors (*n* = 13), (GII) with grade III tumors (*n* = 7), and a control group (*n* = 10). The definition of groups GI and GII was based on the results of the histopathological examination of the breast tumor [24] after surgical resection.

### 2.4. EV Isolation

After blood collection, a period of 15 to 20 min was allowed for clot formation, and a series of 4 centrifugations was started. The first centrifugation (Celm, Ls4, Barueri, São Paulo, Brazil) was at ~720× *g* for 10 min at room temperature. The serum (supernatant) was collected and centrifuged again at 4 °C, 3 times (Eppendorf^®^ 5810r, Hamburg, Germany) to remove cell debris and larger particles (300× *g*/10 min, 2000× *g*/10 min, and 16,500× *g*/30 min) following the protocol by Théry et al. [27]. After the final centrifugation, the supernatant was aliquoted and stored in a freezer at −80 °C for subsequent isolation of extracellular vesicles.

Isolation of EVs was performed by size exclusion chromatography using a commercial chromatography column (qEV original 35 nm Gen. 2, Izon Science LTDA, Christchurch, New Zealand). Briefly, the drip valve was opened to allow the liquid contained in the column to be reduced to near the stationary phase. Next, 15 mL of mobile-phase phosphate buffer saline (137 mmol NaCl, 2.7 mmol KCl, 10 mmol Na_2_HPO_4_, and 2 mmol KH_2_PO without Ca^2+^ or Mg^2+^) was added over the stationary phase for initial washing. In the same way, the PBS dripping was allowed until close to the stationary phase, when 500 mL of the serum sample was added. After dripping the entire sample, another aliquot of PBS was added to continue the separation. After adding the sample to the column, 3 mL were collected and discarded (void), since in this method, the larger particles are the first to pass through the column. Next, 2 mL were collected and stored, as this volume contains the smallest particles.

After collecting the EVs, the columns were washed with 15 mL of PBS, and 20 mL of a 7.7 mmol sodium azide was added and kept at 4 °C to reuse. EV samples were kept on ice until quantification and characterization.

### 2.5. Quantification and Characterization of EVs

For the characterization, concentration, size, and size distribution of the EVs, nanoparticle tracking analysis was used. The size of the EVs was also confirmed by transmission electron microscopy (TEM), as recommended by Ávila et al. [28] and Garnica et al. [29]. Briefly, after the isolation of EVs, samples were diluted 1:20 and 1:4000 in PBS, and an aliquot was used for nanoparticle tracking analysis (NanoSight NS 300, Malvern Panalytical, Malvern, UK). Five 30-s videos were captured at level 12 on the camera, using between 20 and 100 particles per frame and a controlled temperature of 38.5 °C. The captured images were analyzed using specific software (NanoSight NTA 3.4, Malvern Panalytical, Malvern, UK).

For TEM, samples were concentrated by ultracentrifugation at 120,000× *g* for 70 min at 4 °C (Optima XE-90 Ultracentrifuge; 70 Ti rotor; Beckman Coulter, Brea, CA, USA), the supernatant was discarded, and the pellet was resuspended in 50 uL of PBS, followed by the addition of 400 uL of fixative (0.1 mmol sodium cacodylate, 2.5% glutaraldehyde, and 4% paraformaldehyde, pH 7.2–7.4) and incubated for 2 h at room temperature. After incubation, 2 mL of PBS were added, and the sample was ultracentrifuged again at the same speed, time, and temperature. Again, the supernatant was discarded, and the pellet was resuspended in 100 uL of ultrapure water (MilliQ, Merck Millipore, Sao Paulo, Sao Paulo, Brazil). Samples were refrigerated until analysis. For TEM, the solution containing the EVs was placed on a copper grid for 20 min at room temperature, with the addition of 2% uranyl acetate and analyzed in a transmission electron microscope (FEI Tecnai 20; LAB6 emission; 200 kV, Hillsboro, OR, USA).

### 2.6. Preparation of Samples for Mass Spectrometry

After the isolation of EVs, the proteins were prepared for mass spectrometry. To extract proteins from serum EVs, 1.2 × 1010 particles/1.5 mL of buffer were used. Serum EV samples were added to RIPA buffer (150 mmol NaCl, 1% Triton X-100, 1% sodium deoxycholate, and 0.1% SDS in 50 mmol TRIS-HCl pH 7.5) [30] containing protease inhibitors (0.8 mmol EDTA, 1 µg/mL aprotinin, 1 µg/mL leupeptin, and 35 µg/mL PMSF [phenylmethylsulfonyl fluoride] in 50 mmol Tris-HCl pH 7.2) [31]. The samples were sonicated following the protocol proposed by Souza et al. [32], followed by centrifugation at 10,000× *g* for 30 min at 4 °C. After sonication and centrifugation, the samples were concentrated in an ultrafilter (Amicon^®^ 3 kDa, Merck-Sigma-Aldrich, Sant Louis, MO, USA) 3 times at 4 °C (2× at 14,000× *g*/30 min and 1× at 14,000× *g*/10 min), with a final volume of 120 µL. Subsequently, the volume was washed with 50 mmol of ammonium bicarbonate (AmBic, Merck-Sigma-Aldrich, Sant Louis, MO, USA) in the ultrafilter 5 times at 14,000× *g*, for 10 min, at 4 °C, according to the manufacturer’s instructions.

The total protein concentration was determined by the Bradford method using a nano spectrophotometer (NanoDrop™ One/One, Thermo Fisher Scientific, Waltham, MA, USA) based on known concentrations of bovine serum albumin to establish a standard curve. After determining the total protein concentration, the samples were prepared for polyacrylamide gel electrophoresis under denaturing conditions with a 10% separation gel using 25 µg. The run was stopped when the samples reached the separation gel. The gel was fixed (40% ethanol and 10% glacial acetic acid) and stained for 12 h with colloidal Coomassie blue G-250 [33]. The samples were then digested using the protocol proposed by Shevchenko et al. [34], with modifications. Single bands were clipped, and decolorization was performed 5 times with 50% acetonitrile in 25 mmol of ammonium bicarbonate (AmBic). The gel fragments were dehydrated with 100% acetonitrile. The reduction of disulfide bridges was carried out with 20 mmol of DTT in 50 mmol of AmBic and the alkylation with 55 mmol of iodoacetamide in 50 mmol of AmBic. Subsequently, digestion was performed with trypsin (1:25 substrate/trypsin) (Promega Trypsin, Code No V511, São Paulo, São Paulo, Brazil) for 14 h. After this period, trypsin was blocked with 5% formic acid in 50% acetonitrile. The peptides were eluted 3 times: first with 1% formic acid in 60% methanol, followed by 1% formic acid in 50% acetonitrile, and then 100% acetonitrile. The volume of the samples was reduced to ~1 µL (Speed Vac™, SPD1010 Integrated SpeedVac™ Systems, Thermo Fisher Scientific Inc., Waltham, MA, USA) and stored at −20 °C until mass spectrometry.

For mass spectrometry, samples were thawed and diluted in 0.1% formic acid (0.7 μg protein/μL) and centrifuged at 1100× *g* for 5 min. Then, 15 μL of the supernatant was placed in a vial with a screw cap for mass spectrometer (Clear glass 12 × 32 mm, Waters Corporation, Milford, MA, USA). An aliquot of 4.5 μL was separated in a C18 column (100 μm × 100 mm) using nanochromatography (nanoACQUITY UPLC^®^, Waters Corporation, Milford, MA, USA) coupled to a Q-Tof spectrometer (Micromass^®^ Q-Tof PREMIER^®^ Mass 12 Spectrometer, Waters Corporation, Milford, MA, USA), with an electrospray source in a flow of 0.60 μL/min, the voltage maintained at 3.5 kV, a voltage cone of 30 V, and a temperature of 100 °C. The gradient of acetonitrile (2–90%) in 0.1% formic acid was maintained for 45 min. The equipment was operated in the top three modes, in which an MS spectrum was acquired, followed by MS/MS of the 3 most intense peaks detected. After MS/MS fragmentation, the ions were placed on an exclusion list for 60 s.

The search parameters were trypsin as a protease, 1 cleavage allowed, cysteine carboxyamidomethylation as a fixed modification, methionine oxidation as a variable modification, an error tolerance of 1 Da for both mass spectrum (MS) and fragment ion mass (MS/MS), and monoisotopic molecular mass.

Spectra were acquired using MassLynx™ v.4.1 software (Waters Corporation, Milford, MA, USA), and the raw data were converted to a peak list (.mgf) without adding scans and analyzed using Mascot Distiller 2.4.0.0 software (Matrix Science Inc., Boston, MA, USA) to obtain the relative quantification of each protein in the mixture, which was determined by the exponentially modified protein abundance index (emPAI) [35]. The results were searched in the UniProt KB database (www.uniprot.org/ accessed on 6 August 2023) using the taxonomy *Canis lupus familiaris* (UP000002254). The results were analyzed using Protein Pilot 4.0 software (AB Sciex, Framingham, MA, USA) for validation of mass spectra and identification of proteins. The mass spectrometry proteomics data have been deposited to Mendeley Data (https://data.mendeley.com/datasets/rfp7kfjcsd/1 accessed on 9 August 2023) via the repository with the data-set identifier in the csv format [36].

### 2.7. Statistical Analysis

The variables concentration and size of EVs are described as mean ± standard error and analyzed by Sigma Plot software v.11.0. The variables were subjected to the Smirnov–Komolgorov normality test and the Levene test to assess the equality of variances. Comparisons of the size of EVs between groups were conducted by one-way ANOVA, and for multiple comparisons, the Tukey test was used. For statistical analysis of the EV concentration, the Kruskal–Wallis test was used, and for multiple comparisons, the Tukey test. Differences were considered significant at *p* < 0.05.

For proteomics, the data were normalized to exclude proteins that did not represent the population. In this way, proteins that were not present in at least 40% of the samples in each group were considered absent in the group, and if the same situation occurred in all groups, the protein was excluded from the analysis. Outliers were corrected by dividing the emPAI of each protein in the sample by the sum of the emPAIs of the protein in all samples. Non-hierarchical clustering was used as multivariate analysis with MetaboAnalyst 5.0 software (www.metaboanalyst.ca/ accessed on 28 June 2023) [37]. Principal component analysis (PCA) described the sample variation in the score matrix. A heatmap was built to compare the groups. ANOVA was used to determine the difference between groups using Fisher’s LSD test, considering significant values when the false discovery rate (FDR) was <0.05. The analysis was also performed to compare control vs. GI, and control vs. GII, using PCA, dendrogram, and fold change (threshold FC > 2). A ROC curve was built in the MetaboAnalyst to identify a biomarker. Pathview free online software (https://pathview.uncc.edu/analysis_api accessed on 2 August 2023) was used to compare GI vs. control and GII vs. control group and build the GO enrichment [38,39,40].

## 3. Results

The neoplasms were classified histologically to define the groups and are described in Supplementary Tables S1 and S2. The mean ± standard error of the age of the bitches in the control group was 1.95 ± 0.33 years, in the GI was 10.07 ± 0.60 years, and in the GII was 12.10 ± 0.70 years. The mean weight ± standard error values were 16.2 ± 2.4 kg, 10.1 ± 3.6 kg, and 10.9 ± 3.3 kg, for the control group, GI, and GII, respectively.

Owners of female dogs with neoplasia (GI and GII) reported having used progestogens in 5% (1/20) of the animals, 70% (15/20) reported never having used these drugs, and 25% (4/20) were unable to provide this information.

**Figure 1:**
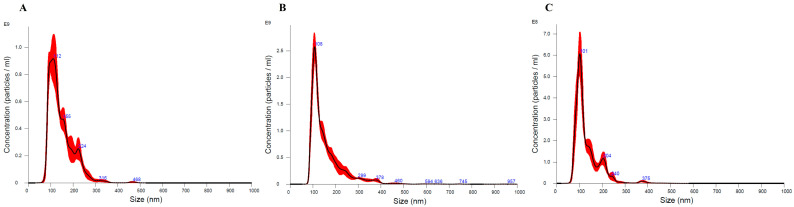
The images represent the EVs size (nm) measured by the nanoparticle tracking analysis (NanoSight NS 300, Malvern Panalytical, Malvern, UK). EVs isolated from control group (**A**), GI (**B**) and GII (**C**) ranging from 30 to 150 nm.

**Figure 2:**
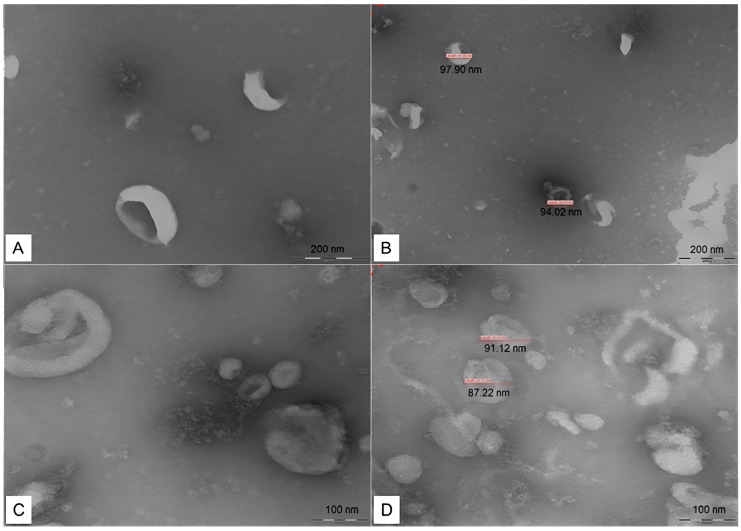
Transmission electron microscopy image of serum EVs isolated from bitches. The particles have spherical/oval shape (**A**,**C**) and measured between 30 and 150 nm (**B**,**D**).

EVs concentrations did not differ between groups (Figure 1). For particle size analysis, the mode was used, with mean ± standard error values in the control group of 136.2 ± 6.0 nm, in the GI of 129.3 ± 4.0 nm, and in the GII of 125.5 ± 6.1 nm. There was also no statistical difference between groups for particle size (*p* = 0.396). In the characterization of EVs in electronic microscopy, spherical/oval-shaped particles were found (Figure 2).

**Figure 3:**
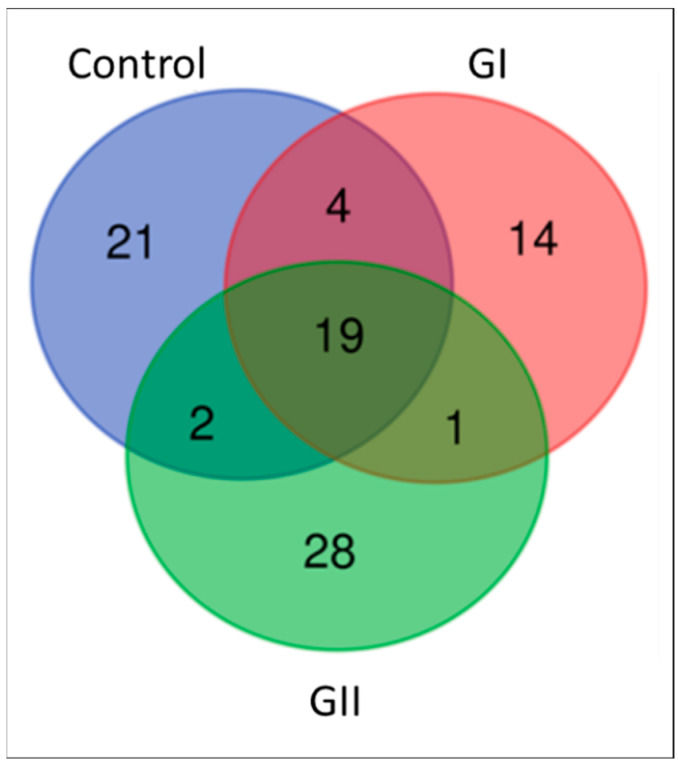
Venn diagram presenting the proteins found in control group, GI, and GII.

**Figure 4:**
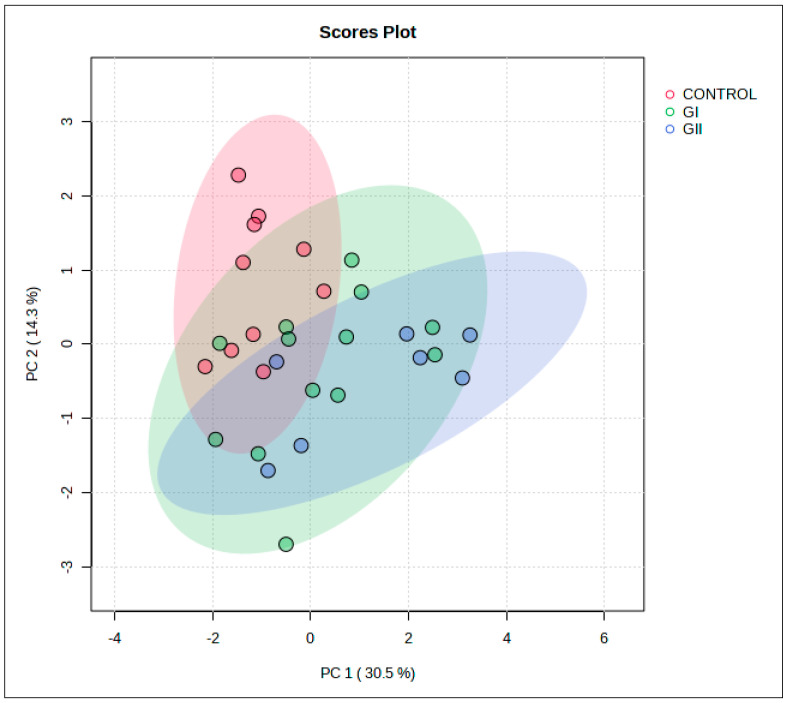
Principal component analysis (PCA) of the multivariate statistical from the control group, GI, and GII.

**Figure 5:**
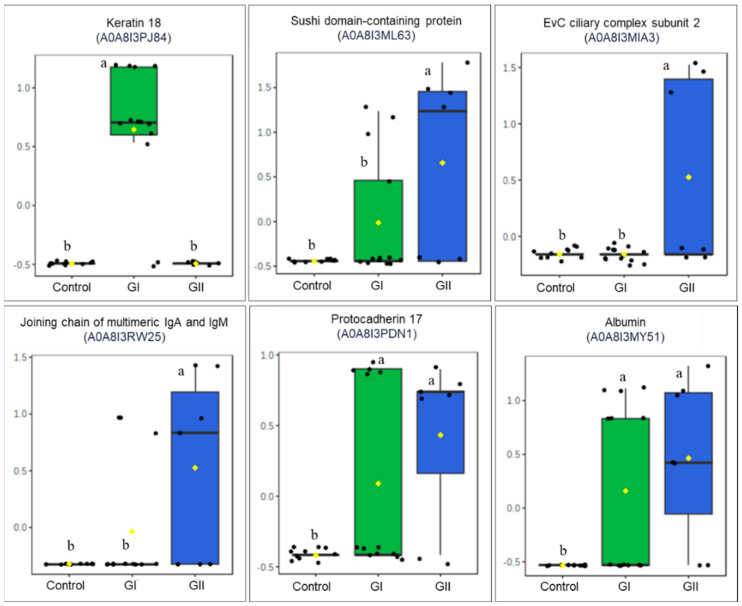
Differential abundance of proteins in control group, GI, and GII. Different letters (a and b) indicate significant statistical difference (ANOVA–Fisher’s LSD test, FDR < 0.05.). ^ab^ Different letters indicate statistical difference (*p* < 0.05).

In mass spectrometry of EVs, 91 proteins were identified, with different numbers for each group (Figure 3). Supplementary Tables S3 and S4 of the Supporting Information describe the results obtained for each group, including the molecular function, biological process, and cellular component of each protein. The PCA indicated an overlapping of groups, with emphasis on the control group (Figure 4), and 44.8% (PC1 + PC2) variation was explained. The results of univariate analysis (ANOVA) indicated 6 proteins with different abundances between groups (FDR < 0.05, Figure 5). The keratin 18 protein (A0A8I3PJ84) was the most abundant in GI, while the most abundant proteins in the GII were the sushi domain-containing protein (A0A8I3ML63), the EvC ciliary complex subunit 2 (A0A8I3MIA3), and the joining chain of multimeric IgA and IgM (A0A8I3RW25). The proteins protocadherin 17 (A0A8I3PDN1) and albumin (A0A8I3MY51) were the most abundant in both groups composed of animals with neoplasms (GI and GII).

**Figure 6:**
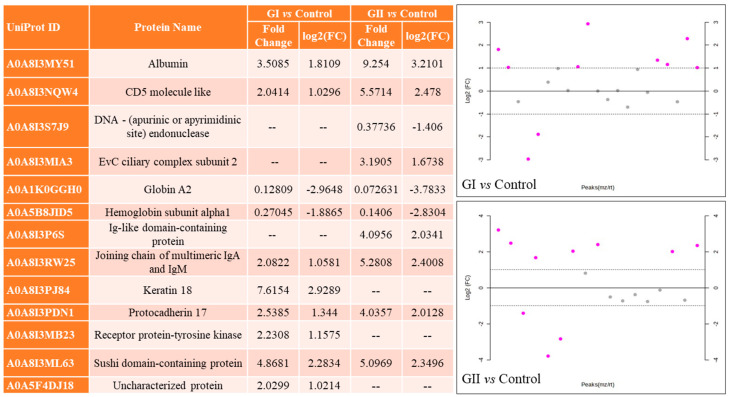
Results of the fold change analysis for comparing GI or GII vs. control. Purple dots indicate proteins that were upregulated or downregulated.

**Figure 7:**
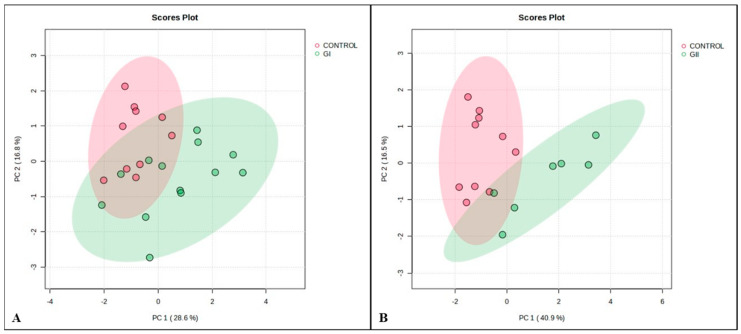
Principal component analysis (PCA) for GI vs. control (**A**; PC1 + PC2 = 45.4) and GII vs. control (**B**; PC1 + PC2 = 57.4).

In the fold change (Figure 6) analysis, 13 proteins were found in different abundances when comparing the control group with GI or GII, with 7 being common to the 2 groups of bitches with neoplasia (Figure 7A). The PCA results indicated group separation considering the protein profile, since the total values of PC1 + PC2 were 45.4 and 57.4, respectively, for GI and GII (Figure 7B).

**Figure 8:**
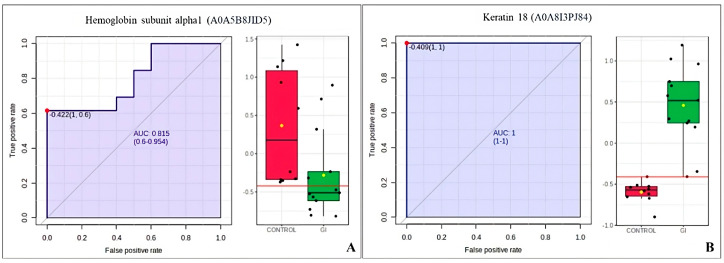
ROC curve of the GI vs. control group from bitches with mammary tumors. (**A**) Hemoglobin subunit alpha1 (A0A5B8JID5). (**B**) Keratin 18 (A0A8I3PJ84).

**Figure 9:**
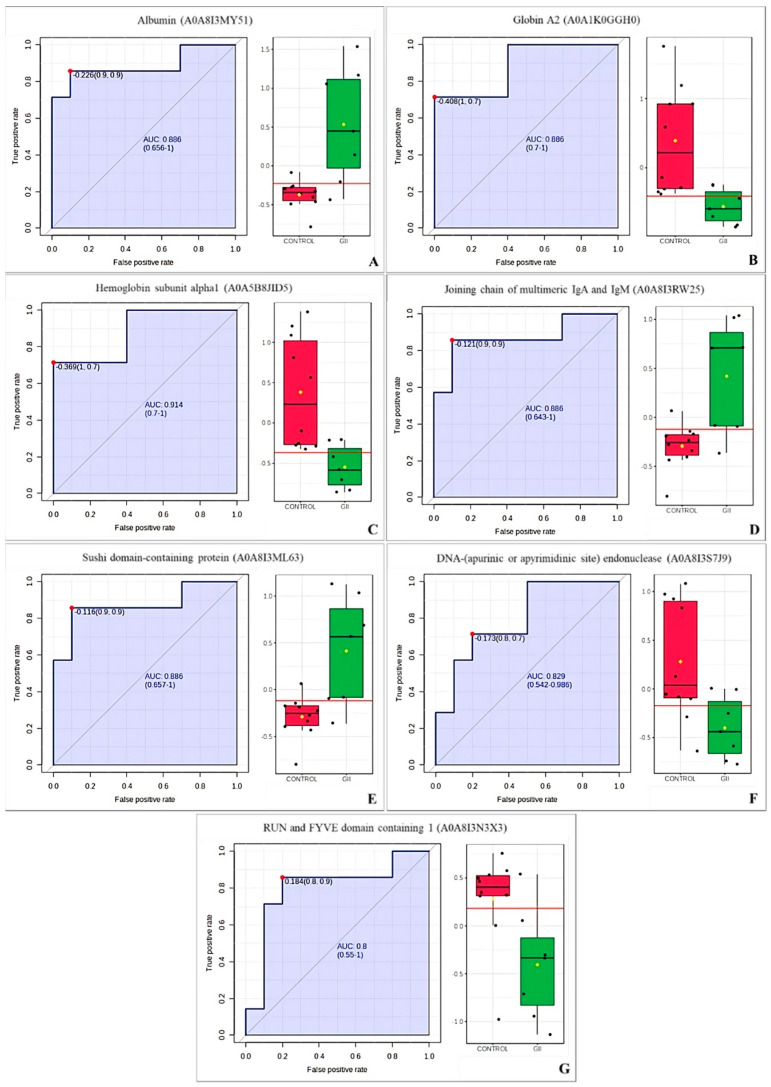
ROC curve of the control group vs. GII from bitches with mammary tumors. (**A**) Albumin (A0A8I3MY51). (**B**) Globin A2 (A0A1K0GGH0). (**C**) Hemoglobin subunit alpha1 (A0A5B8JID5). (**D**) Joining chain of multimeric IgA and IgM (A0A8I3RW25). (**E**) Sushi domain-containing protein (A0A8I3ML63). (**F**) DNA-(apurinic or apyrimidinic site) endonuclease (A0A8I3S7J9). (**G**) RUN and FYVE domain containing 1 (A0A8I3N3X3).

**Figure 10:**
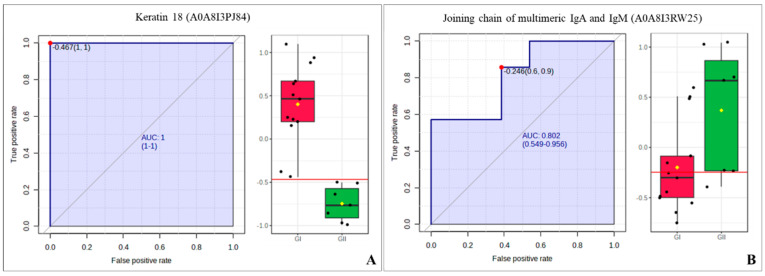
ROC curve of the GI vs. GII from bitches with mammary tumors. (**A**) Keratin 18 (A0A8I3PJ84). (**B**) Joining chain of multimeric IgA and IgM (A0A8I3RW25).

We investigated biomarkers using ROC curve analysis to compare the tumor groups (GI or GII) vs. control group. In the GI vs. control group analysis, 2 proteins were found with an AUC > 0.80, the hemoglobin subunit alpha1 (A0A5B8JID5) and keratin 18 (A0A8I3PJ84) (Figure 8); while in the GII vs. control group, 7 proteins had an AUC > 0.80, albumin (A0A8I3MY51), globin A2 (A0A1K0GGH0), hemoglobin subunit alpha1 (A0A5B8JID5), joining chain of multimeric IgA and IgM (A0A8I3RW25), sushi domain-containing protein (A0A8I3ML63), DNA-(apurinic or apyrimidinic site) endonuclease (A0A8I3S7J9), and RUN and FYVE domain containing 1 (A0A8I3N3X3) (Figure 9). Two proteins had an AUC > 0.80 for the GI vs. GII comparison: keratin 18 (A0A8I3PJ84) and the joining chain of multimeric IgA and IgM (A0A8I3RW25) (Figure 10).

**Figure 11:**
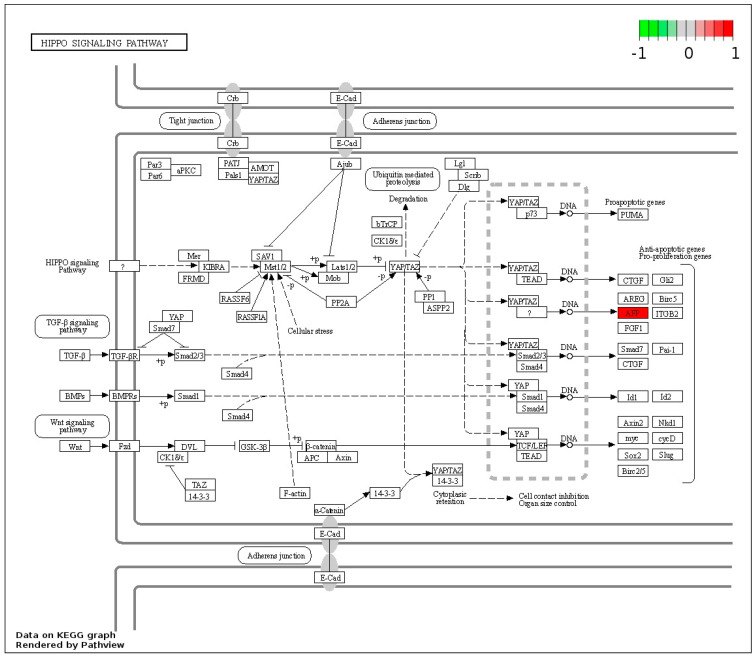
Enrichment pathway of the GI compared to control. AFP gene highlighted in red indicates upregulation in GI (generated in https://www.genome.jp/kegg/).

The enrichment pathway showed that the AFP gene was upregulated in the GI (Figure 11).

## 4. Discussion

The current study aimed to investigate the EVs in the blood serum of bitches affected by mammary neoplasia and their association with the malignancy of the disease, in order to propose serum biomarkers, or suggest likely therapeutic targets.

The animals were divided into subgroups according to the degree of differentiation of the most aggressive neoplasms, grouping the histological subtypes with the most similar characteristics [24,26]. The high number of subtypes is a critical factor in studies of mammary neoplasms in female dogs, since it is difficult to standardize the results [24,41]. Mammary neoplasms mainly affect women over 55 years of age and middle-aged to older dogs [1,42]. In bitches, risk factors, including age, reproductive status, and genetics, seem to predispose to neoplasia in certain dog breeds [3]. In the current study, the average age of female dogs affected by neoplasms followed the results described in the literature, with a higher occurrence in animals aged between 8 and 10 years [1,2,3].

The average weight of female dogs with mammary neoplasms can vary according to the population being studied [43,44]. Our results for the average weight of Groups I and II were 10.1 and 10.9 kg, respectively; however, a limitation of the study was the lack of an individual classification of the body condition score, which could determine the body condition of bitches as thin, adequate weight, overweight, or obese. This parameter may be important but is not frequently mentioned in studies with animals. In women, a higher risk of recurrence, unaffected treatment, and a higher chance of surgical complications were observed due to overweight and obesity [45,46]. Overweight and obesity can be considered risk factors in the development and progression of breast cancer in women and dogs; however, the physiological mechanism has not yet been fully determined. It is suggested that adipose tissue accumulation and the exposure of mammary tissue to growth factors and hormones such as estrogen and progesterone could predispose to tumors, especially in those with a higher degree of malignancy and a worse prognosis [47,48]. Adipose tissue produces pro-inflammatory factors that are associated with the development of neoplasms [49]. In another study, overweight or obese bitches were diagnosed with mammary tumors at a younger age compared to bitches classified as thin or with adequate weight. In addition, they had a more significant lymphatic infiltration by neoplastic cells, which could be a predisposing factor to the occurrence of metastases [47].

Exogenous hormones, especially progestogens, used for estrus suppression, are associated with the development of mammary neoplasm due to improper use and long-acting presentations [50,51]. In our study, only 1 of the 20 owners (5%) of the bitches with mammary neoplasms declared having used progestogens to suppress estrus in the past; however, 25% (4/20) were unable to inform us about the previous use of hormones for this purpose. The use of progestogens to suppress the estrous cycle and prevent undesirable pregnancies is commonly observed in small animal practices [52]. The lack of information to the owner about the appropriate use of these hormones, as well as the adverse effects, are the main problems faced in reducing the use of these drugs. In our study, we did not perform statistical analysis to verify whether there was a relationship between the use of progestogens and the development of breast cancer. However, given the relationship between breast tumors and endogenous and exogenous steroid hormones [50,53], inquiring about the exogenous use of hormones should be considered during the anamnesis of patients with breast nodules, as well as receiving clarification from the owner regarding its use and adverse effects.

Given the importance of mammary tumors in female dogs, serum or plasma EVs have shown promise in the pursuit of minimally invasive methods that seek to identify biomarkers that may aid monitoring of mammary neoplasms [20,22,54]. In our study, to validate the EV isolation method, we used the concentration and characterization by size and shape of the EVs, which were performed by nanoparticle tracking analysis (NTA) and transmission electron microscopy (TEM) [27,55,56]. The NTA analyzes the EV distribution by quantity and size, using the Brownian motion of particles in suspension [57]. Regarding particle size, our results were similar to those described in the serum EVs of humans [11] and animals [19,29]. Furthermore, according to the International Society of Extracellular Vesicles (ISEV), these particles can be classified as small, ranging from 30 to 150 nm [58].

The concentration of EVs/mL of blood serum in bitches with mammary neoplasms showed no difference from the control group. EVs are secreted by different cell types; moreover, their circulating concentration may be influenced by several factors, such as sample handling and storage, pH of the secreting cell, cell hypoxic and death, oxidative stress, individual alterations, the patient’s clinical status, cancer, and inflammation. These aspects were previously related to an increase or decrease in the EV concentration in tissues and fluids [59,60,61,62]. Although several studies have investigated these nanoparticles, the concentration range of EVs in body fluids such as blood serum has not yet been specified [63]. The absence of systemic disorders in the group of animals selected for our study could justify the similar results between the groups, which were also found in another study of serum EVs in women with breast cancer [64].

The results are conflicting regarding the increase, decrease, or absence of differences in EV concentrations in cancer patients. In women with breast cancer, studies did not observe differences in the concentrations of EVs in blood serum when compared to patients without cancer [64]. Other studies found an increase in the concentration of EVs in humans with hepatocellular carcinoma compared to the control group or neoplastic groups of different stages [65]. These findings were correlated with greater secretion of EVs by tumor cells, tumor aggressiveness, and prognosis [66].

In veterinary medicine, there is also no definition regarding the concentration of EVs in animals with neoplasia. A study observed individual differences between patients with or without cancer, but without statistical significance [67]. Other workers demonstrated an increase in EVs derived from platelets, leukocytes, and T lymphocytes in blood plasma from animals with different types of neoplasms [20]. Concentration differences of EVs in the blood serum of dogs with lymphoma were also related to the response to chemotherapy; patients in remission had lower concentrations of EVs than animals with an unsatisfactory response to chemotherapy or with progression in the disease [29].

The shape of the EVs was analyzed by TEM. This technique was chosen due to its practicability, reduced cost, and wide use in studies with EVs [55]. Spherical/oval-shaped particles were observed, similar to those described in studies with the same type of biofluids [67] and species [54]. However, studies also indicate interference in the process of fixing the slide for reading the sample in the TEM, which can discreetly alter the spherical shape of the EVs. Therefore, another method for the characterization of the format is cryo-electronic microscopy, since this technique allows 3D visualization of the nanoparticles, with less interference from the fixation medium on the sample [27,56,57,68].

Proteins are macromolecules composed of a set of amino acids that are important for the structure and performance of cellular functions. EVs contain information about their cells of origin; thus, proteins can play a key role in identifying biomarkers that can help clarify the biological processes involved in carcinogenesis [7,69]. The CD5-like-molecule protein (A0A8I3NQW4) was identified in all groups in our study, however, with no differences between groups. This protein, which is secreted with EVs [58], was identified in blood serum samples [11] and suggested as a biomarker of EVs isolated from blood plasma in humans [70,71]. This CD5-like gene was also identified in women with breast cancer [72] and in dogs with leishmaniasis [73]. Therefore, in our study, mass spectrometry was considered an additional method for characterization and validation of the isolation of EVs, in addition to the NTA and MET.

In the proteomics results, the ROC curve analyses highlighted three proteins: hemoglobin subunit alpha (A0A5B8JID5), which was decreased in GI and GII; keratin 18 (A0A8I3PJ84); and joining chain of multimeric IgA and IgM (A0A8I3RW25), which were increased in the GI and GII.

Keratin 18 (A0A8I3PJ84) is a type I cytoskeletal protein, an intermediate filament with structural function, expressed in single-layer epithelial tissues, and can therefore be considered an epithelial marker [74]. This protein is usually secreted by cells undergoing apoptosis and can also be used as a serum marker of cell death [74,75]. It is the object of combined investigations using proteomics and immunohistochemistry techniques, in the pursuit of biomarkers for breast neoplasms, where, primarily, a relationship is observed between the expression of cytokeratins and the epithelial origin of this neoplasm and the relationship between the high expression of keratin 18 and the lower invasiveness of feline mammary carcinomas [76]. This protein has already been suggested as a biomarker of large oncosomes, which are EVs derived from cancer cells with a diameter between 1000 and 10,000 nm and that have a different protein profile. The same study used western blotting to demonstrate a higher amount of keratin 18 in EVs derived from the plasma of patients with prostate cancer, compared to healthy patients [77]. In our study, keratin 18 showed greater abundance in GI compared to control and GII, thus suggesting it as a potential regulator of the tumor growth process, since studies indicate that a lower abundance of this protein in patients with cancer in advanced stages is related to greater tumor invasiveness and consequently a worse prognosis [78]. It is suggested that the lower abundance in GII, a group with higher-grade neoplasms, may be related to an aberrant methylation of the KRT18 gene [79], thus causing silencing of this gene and lower expression of the protein in this group. The lower abundance of the keratin 18 protein in cell lines of breast cancer in humans has already been related to partial induction of the mesenchymal-epithelial transition (MST) by increasing EpCam expression and activation of the Wnt/β-catenin pathway, promoting an increase in metastatic characteristics of mammary neoplasms [80]. However, more studies are needed to prove this hypothesis in mammary neoplasms in bitches. Intriguingly, this protein was considered a biomarker for GI in our study in the ROC curve analysis. Thus, keratin 18 could be an important marker of disease progression.

In GII, the most abundant proteins were sushi domain-containing protein (A0A8I3ML63), EvC ciliary subunit 2 (A0A8I3MIA3), and joining chain of multimeric IgA and IgM (A0A8I3RW25). The sushi domain-containing protein is a protein homologous to complement component-binding 4 protein alpha (C4BPA—A0A8C0P5M3). C4BPA is involved in the innate immune response through activation, fat metabolism, and regulation of the complement system, a system that has been studied for its role in carcinogenesis [81,82]. This protein has previously been identified, without significant difference, in serum from healthy dogs with mammary neoplasms [83] and obese dogs, being more abundant in animals with metabolic disorders [81]. In EVs, human studies have already identified C4BPA in serum EVs from patients with melanoma [84]. In EVs derived from blood plasma, a correlation between C4BPA, modulation of the tumor microenvironment, and immune escape through the complement system has been suggested in patients with hepatocellular carcinoma, showing a higher abundance of C4BPA compared to control patients [85]. This hypothesis of a protection effect and tumor escape by the complement system can be suggested by our results, since the group with neoplasms of higher grade and complexity had a greater abundance of the homologous protein sushi domain-containing protein. However, further analyses are needed to confirm this hypothesis in mammary neoplasms in bitches.

EvC ciliary subunit 2 is a transmembrane protein that plays a role in biological processes, embryonic development, and bone and skeleton formation [86]. Mutations in the gene for this protein can cause so-called ciliopathies, such as Ellis–van Creveld syndrome in humans, also known as chondroectodermal dysplasia [87,88]. Another consequence of gene mutations is that they promote dysfunctions in hedgehog signaling. This signaling pathway is suggested to be involved in several stages of carcinogenesis and as a therapeutic target, but the mechanism is not yet fully understood [89,90,91]. A study with breast tumors found significant mutations in the EVC2 gene, but in the metastases and not in primary tumors, thus suggesting targets for metastasis inhibition [92]. This gene has also been identified with increased methylation in cells of triple-negative breast cancer in patients who were considered non-responsive to neoadjuvant chemotherapy [93]. Higher methylation in a gene suggests lower synthesis and, consequently, a lower abundance of this protein [94]. However, our results showed a higher abundance of the EvC ciliary subunit 2 protein in the GII group, different from the results proposed in the literature.

The joining chain of multimeric IgA and IgM is a protein with ~15 kDa, which connects IgA and IgM monomers to form multimeric IgA (dimer) and IgM (pentametric) antibodies. It is transported by the epithelium and has a protective function on the mucosal surface, mediating the immune response and inflammation [95,96]. Moreover, in a study that investigated different origins of EVs, such as tissue, plasma, and serum, this protein was identified in human EVs associated with other proteins, whose function is endocytosis and exocytosis markers [97]. In a cohort study, higher expression of mRNA of the JCHAIN gene was correlated with favorable survival in women with breast cancer of luminal origin and proposed as a possible biomarker [98]. This protein has already been identified in serum and saliva samples from dogs with mammary neoplasms, with no difference in abundance between groups with and without neoplasia [83]. In our study, the joining chain of multimeric IgA and IgM was detected in higher abundance in GII compared to the control group and GI and was associated with an AUC > 0.80, indicating that the protein could be suggested as a biomarker of malignancy of mammary cancer in dogs.

The groups with mammary neoplasms (GI and GII) showed a higher abundance of the proteins protocadherin 17 and albumin. Protocadherin 17, encoded by the PCDH17 gene, is a type I transmembrane protein of the cadherin subfamily. In prostate cancer in men, the exacerbated methylation of the gene encoding this protein, obtained by PCR in tissue, was related to a higher pathological grade, lymph nodes with metastases, and a lower survival rate, with PCDH17 hypermethylation being suggested as a possible biomarker of this neoplasm [99]. In women with breast cancer, this protein was more abundant in neoplastic tissue with bone metastasis compared to non-metastatic tissue, promoting cell invasion [100]. On the other hand, studies have shown a lower expression of the PCHD17 gene in neoplastic tissues, correlated with a greater abundance of markers of angiogenesis and hypoxia in the serum of these patients [101], and increased ectopic expression of PCDH17 promoted inhibition of cell proliferation and mobility [102]. In dogs with diffuse large B-cell lymphoma, a lower expression of the PCDH17 gene was also observed in the cell culture of this neoplasm, which was related to the hypermethylation of this tumor suppressor gene [94]. The literature contains conflicting results, as presented above, so we suggest that the higher abundance of PCDH17 in the neoplasia groups from our study could be due to the heterogenicity of this protein in EV-enriched samples from tissue and serum samples, and given its role in women with breast cancer, this protein should be further investigated and validated in serum and tissue from bitches with a mammary tumor, in order to improve the accuracy of these findings.

Albumin (A0A8I3MY51) is the most abundant protein in serum, which is considered a co-isolation in EV-enriched samples and has been previously reported [58]. If we consider albumin as a possible contaminant of the samples, it is suggested that this parameter should not differ between the groups, since the methods of isolation and processing of the samples were similar in all groups. In addition, a study demonstrating the use of size exclusion chromatography for EV isolation reported lower co-isolation of this protein when compared to ultracentrifugation and precipitation methods [103]. However, in our study, it was observed that the animals in the groups with neoplasms had a greater abundance of this protein.

Although in studies with EVs from blood serum, albumin is considered a contaminant [104], there are descriptions of the functions of EVs induced by albumin, such as the polarization of M129 macrophages and increased uptake of EVs, influencing communication between cells and the cellular environment [105]. The uptake of EVs loaded with albumin in macrophage cells and breast cancer cells indicates an efficient and uniform uptake of albumin by the cells. These results suggest that loading albumin into EVs may be a promising strategy for directing albumin delivery to specific cells, such as macrophages and breast cancer cells, and may have significant implications for targeted protein delivery and future therapeutic applications [106].

Albumin, which has a molecular weight of ~66 kDa, is synthesized by the liver and has functions in cellular metabolism and the transport of endogenous and exogenous compounds [107]. This protein was found in lower abundance in mass spectrometry [83] and a lower free concentration in the blood serum of animals with mammary neoplasms. In addition, a lower abundance of animals with metastases and concomitant diseases was found in healthy patients [83,108]. Moreover, in the pathway graph data analysis, we found the AFP gene, which translates alpha-fetoprotein, which has a 90% identified sequence to albumin (UniprotDB). Alpha-fetoprotein is a fetal analog of serum albumin and is a related protein [109].

Low albumin concentration in patients with metastatic breast cancer was positively correlated with the probability of lower cancer-specific survival due to the presence of systemic inflammation and the patient’s frailty, both factors that can influence the progression of the disease and the response to treatment [110]. In our study, there was a higher abundance of albumin in the proteomics of serum EVs in GII, which may not be just a contaminant as a difference was observed between the control and GI groups.

Regulation of breast cancer cell proliferation and migration occurs due to complex interactions between signaling pathways and proteins that mediate the hippo-pathway [106,111,112], controlling the activity of Yes-associated protein (YAP) and transcriptional co-activator with PDZ-binding motif (TAZ), key components of the hippo pathway. YAP regulates cell proliferation, while TAZ regulates cell migration [113], promoting epithelial-mesenchymal transition, invasion, migration, cancer stem cell-like characteristics, tumor growth, and metastasis in breast cancer cells [112]. This scenario suggests perspectives for the development of therapeutic approaches targeting this pathway in breast cancer. In our study, it was observed that activation of this pathway increased alpha-fetoprotein (AFP) levels.

Specific receptors for AFP in the membrane of human breast cancer cells were indicated as a marker of cancer cells in clinical blood samples from patients with breast carcinoma [114]. The chimeric alpha-fetoprotein peptide, modified and created from AFP [115], was investigated as an anticancer agent in the treatment of breast cancer, particularly estrogen receptor-positive breast cancer, and resulted in alterations in the aminoacyl-tRNA, glycolysis, gluconeogenesis, and biosynthesis of biotin and amino acids, acting on specific cancer cells, with potential as medicine for the treatment of breast cancer [116]. This protein has recently been suggested as a biomarker for diagnosing breast cancer in women [117].

Hemoglobin subunit alpha 1 (A0A5B8JID5) was found with an AUC > 0.80 in the ROC curve and was in lower abundance in the bitches with mammary tumors in our study. This protein was mentioned in lower expression in the plasma from patients with esophageal squamous cell carcinoma [118] and pancreatic cancer [119]. In a study on breast carcinoma, the hemoglobin subunit beta was only seen in normal tissues [120]. In contrast, hemoglobin subunits alpha and beta were found in increased abundance in the plasma of women with breast cancer chemoresistance [121]. Our findings should be interpreted with caution, and the clinical relevance in bitches with mammary cancer needs to be investigated.

The hypoxia detected in neoplasms results from a combination of a high demand for oxygen from the cancer cells, intense cell multiplication, and an insufficiency in the oxygen supply resulting from issues in the vascularization of the tumor [122]. This hypoxic condition is associated with breast cancer, which intensifies the metastatic behavior of this tumor [123,124]. Neoplastic cells adapt to the hypoxic microenvironment [125], and this was correlated with the increase in the release of EVs in breast tumors [126,127]. The expression of globins in solid breast tumors may be related to responses to oxygen supply in tumors [128]. In the current study, globin A2 (A0A1K0GGH0) and hemoglobin subunit alpha1 (A0A5B8JID5), both related to oxygen transport [128,129], were found with an AUC > 0.80 on the ROC curve and with lower abundance in the serum EVs of bitches from GII.

The protein DNA—(apurinic or apyrimidinic site) endonuclease (APEX2)—is crucial in repairing DNA damage, especially at apurinic or apyrimidine sites (AP), where a nitrogenous base is missing [130]. Its main function is to hydrolytically cleave adjacent phosphodiester structures, creating single-strand breaks with 5′-deoxyribose phosphate and 3′-hydroxyl to facilitate repair [131]. APEX2, along with other enzymes, assists in restoring these sites and guarding against oxidative damage [132], which often causes APs, and has a critical role in the base excision repair (BER) mechanism [133]. In summary, APEX2 is important to maintain genomic integrity and prevent genetic mutations [130,131,132,133].

In our findings, the APEX2 protein was found to be reduced in the serum extracellular vesicles of bitches in GII compared to the control group, different from the results found in the meta-analysis conducted by Jensen, Shi, and Yan [134], who identified a higher abundance of this protein in human tumor tissue compared to non-malignant tissue in kidney, breast, lung, liver, and uterus cancers, but not in prostate cancer. The decrease in this protein shown by the ROC curve when comparing GII and the control group indicates an important factor to be considered in cancer progression and response to treatment due to its function in the maintenance of genomic integrity and the prevention of genetic mutations. Further studies are needed for a better understanding of the precise function of this protein in canine mammary tumors.

The RUN and FYVE domain-containing 1 (RUFY1) protein regulates vesicle trafficking and cell membrane dynamics. It has two distinct functional domains: the RUN [135] domain interacts with proteins from the Rab family [136,137], thus assisting the movement of vesicles and organization of the cell membrane, and the FYVE domain, selectively binds to phosphatidylinositol 3-phosphate (PI3P) in the membrane, allowing RUFY1 to be targeted to specific membrane locations [137,138].

The RUFY1 protein is suggested as a biomarker of epithelial-mesenchymal transition and has relevance to the immune system in the metastasis of breast cancer in humans. Furthermore, this protein aids in endocytosis regulation and the distribution of proteins in the plasma membrane [135]. Hypermethylation of sites close to the RUFY1 gene is associated with mammographic density and may be an important epigenetic signature related to breast cancer risk [139]. Gene silencing of RUFY1 expression in gastric cancer cells was associated with its progression [140]. In the present study, this protein was indicated by the ROC curve as a biomarker in the proteomics of serum extracellular vesicles in bitches and was found to be reduced in GII in comparison to the control group. The decrease in RUFY1 in this context may indicate a greater risk of cancer aggressiveness and metastasis, affecting disease progression. Furthermore, this decrease may have implications for the immune response to cancer. However, further research is needed to fully understand the impact of this discovery and its clinical relevance.

In our results, EvC ciliary subunit 2, joining chain of multimeric IgA and IgM, protocadherin 17, and albumin proteins were identified in greater abundance in the neoplasia groups of samples enriched with serum EVs; however, it is possible to observe that in most studies, these proteins and their coding genes are generally found in lower abundance and expression when studied in neoplastic tissues, free in blood serum, and in tumor-derived EVs [83,93,98,102]. EVs carry information on their cell of origin; however, when investigated in the blood, it is possible to suggest that there is heterogeneity in the origin of populations of EVs since they will be secreted both by tumor cells and by cells with other origins, such as leukocytes and platelets [141,142], which is also observed in animals [20]. Therefore, we suggest that the profile and protein abundance found in serum EVs may differ from the findings in neoplastic tissues and in EVs derived from these tissues, as also proposed in humans when comparing the protein profiles of plasma EVs and EVs derived from neoplastic tissue [97]. More comparative studies between serum EVs and tissue-derived EVs are needed to clarify the mechanisms involved in these protein profile differences.

It is important to emphasize that the keratin 18 and protocadherin 17 proteins and their coding genes play an important role in the suppression of proliferation and tumor growth in breast cancer in women [78,79,101,102]. The identification of these proteins in our study highlights that these proteins also seem to play roles in the biological processes involved in mammary tumors in bitches, thus suggesting that they can be considered targets for future studies of mammary neoplasia in canines.

## 5. Conclusions

We conclude that the heterogeneity of breast cancer subtypes is one of the limiting factors for studies with this type of cancer, as it makes it difficult to standardize methodologies as well as findings. The results of the isolation and characterization of EVs agree with what was previously observed in the literature for this type of biological sample and add data to the veterinary literature for the description and characterization of nanoparticles. However, the results referring to EVs did not indicate a relationship with the type and classification of the tumor and cannot be suggested to aid in the diagnosis and monitoring of bitches affected by mammary neoplasms. We suggest that keratin 18 and protocadherin 17 should be further investigated in bitches because of their role in women’s breast cancer. The proteins identified in our study could help to clarify some functions and suggest the possible invasive behavior of mammary neoplasms in bitches, thus being an important advance in the investigation of this type of tumor in veterinary medicine.

## Data Availability

Available in “Mendeley Data” at https://data.mendeley.com/datasets/4kgtd7tnv3/1 accessed on 9 August 2023.

## Supplementary Materials

The following supporting information can be downloaded at: https://www.mdpi.com/article/10.3390/cimb46070459/s1, Tables S1 and S2: Classification of neoplasms to define groups; Table S3: Proteins identified in the different groups; Table S4: Gene ontology analysis of molecular functions, biological processes, and cellular components.
