# Supplementary material for: Serum Extracellular Vesicles Cargo Approach in Bitches with Mammary Tumors
Source: Curr Issues Mol Biol. 2024 Jul 22;46(7):7745–68. doi: 10.3390/cimb46070459 (PMC11275879; doi:10.3390/cimb46070459)
Supplement: Supplementary file 1 [file cimb-46-00459-s001.zip › cimb-3078322-supplementary.pdf]

Approach of Serum Extracellular Vesicles Cargo in Bitches with Mammary Tumors

Gabriela C. Sousa, Marcos G. Carvalho, Carlos E. Fonseca-Alves and Fabiana F. Souza

Supplementary Materials

Table S1. Classification of neoplasms to define groups 2
Table S2. Classification of neoplasms to define groups 4
Table S3. Proteins differentially expressed in groups 5
Table S4. Gene ontology Data obtained of UniprotKB.. 9

Table S1. Breed, reproductive status, histopathology classification, and cancer staging from bitches allocated in GI (n = 13).

Breed	Reproductive status	Histopathology	Stage
**Mixed breed**	Spayed	Carcinoma in mixed tumor grade I<br>Carcinoma in mixed tumor grade I<br>Complex carcinoma grade I	I
**Shi-Tzu**	Spayed	Carcinoma in mixed tumor grade I ectasia ductal	I
**Dachshund**	Spayed	Carcinoma suggesting mixed benign<br>Mixed benign<br>Complex Adenoma	II
**Mixed breed**	Spayed	Carcinoma tubular grade II	II
**Lhasa-Apso**	Intact	Complex carcinoma grade I<br>Carcinoma in mixed tumor grade I<br>Carcinoma in mixed tumor grade II	III
**German Shepard**	Intact	Carcinoma in mixed tumor grade II	III
**Labrador**	Spayed	Complex carcinoma grade I	III
**Mixed breed**	Intact	Complex carcinoma grade I<br>Tubular carcinoma grade I	III
**Mixed breed**	Intact	Complex carcinoma grade I<br>Complex carcinoma grade II	III
**German Shepard**	Intact	Carcinoma in mixed tumor grade I<br>Complex carcinoma grade II	IV
**Poodle**	Spayed	Lobular hyperplasia<br>Lipid-rich carcinoma	IV
**Mixed breed**	Intact	Complex carcinoma grade I	IV

		Carcinoma in mixed tumor grade I	
**Lhasa**-A**pso**	Spayed	Tubulopapillary carcinoma intraductal grade I<br><br>Complelx carcinoma grade I<br><br>Carcinoma micropapillary grade I<br><br>Complex adenoma associated with mastitis	IV

Table S2. Breed, reproductive status, histopathology classification and cancer staging from bitches allocated in GII (n = 7).

Breed	Reproductive status	Histopathology	Stage
**Cocker Spaniel**	Spayed	Comedocarcinoma<br>Carcinoma-micropapillary invasive grade I<br>Complex carcinoma grade I	I
**Lhasa apso**	Spayed	Solid carcinoma grade II	III
**Mixed breed**	Spayed	Mixed carcinoma and solid grade II<br>Adenoma associated with epitheliosis and squamous metaplasia and areas of solid carcinoma grade I<br>Complex Adenoma	III
**Dachshund**	Spayed	Carcinoma and malignant<br>Myoepithelioma grade II<br>Tubular adenoma<br>Complex adenoma	IV
**Maltese**	Spayed	Complex Carcinoma grade II<br>Carcinoma cribriforme grade II	IV
**Mixed breed**	Intact	Solid carcinoma grade II<br>Solid carcinoma grade III	IV
**Australian cattle dog**	Intact	Mammary osteossarcoma<br>Tubulopapillary carcinoma	IV

Table S3.
Proteins extracted from serum extracellular vesicles in dogs 1066 in the GC, GI and GII groups.

**Protein Name**	**ID**	**Gene**	**Group**		
			**Control**	**GI**	**GII**
Acetyl-CoA acyltransferase 2	A0A8I3NU61	ACAA2		X	
A-kinase anchoring protein 3	A0A8I3Q3V5	AKAP3			X
Albumin	A0A8I3MY51	AFP	X	X	X
Apolipoprotein E	P18649	APOE	X		
Asparagine--tRNA ligase	A0A8I3MX22	NARS2			
Beta 3-glucosyltransferase	A0A8I3PAR3	B3GLCT	X		
Cardiotrophin like cytokine factor 1	A0A8I3N6Q7	CLCF1	X		X
Cupin superfamily member 1	A0A8I3MM27	C1H18orf54	X	X	X
C2 domain containing 3 centriole elongation regulator	A0A8I3MYR6	C2CD3			X
CD5 molecule like	A0A8I3NQW4	CD5L	X	X	X
Clock circadian regulator	A0A8I3NBB7				
C-type lectin domain-containing protein	A0A8I3PY16	CLEC1B			X
DNA-(apurinic or apyrimidinic site) endonuclease	A0A8I3S7J9	APEX2	X	X	
DNA-directed RNA polymerase subunit beta	A0A8I3NPE6	POLR2B			X
Double-strand break repair protein	A0A8I3P7Y4	MRE11	X		
Dynamin 1 like	A0A8I3P3R6	Dynamin 1 like	X		
Dynein axonemal heavy chain 2	A0A8I3PUB4	Dynein axonemal heavy chain 2		X	
Dystonin	A0A8I3NP68	DST			X
E3 ubiquitin-protein ligase	A0A8I3NIE2	DTX3	X		
EvC ciliary complex subunit 2	A0A8I3MIA3	EVC2	X	X	X
Family with sequence similarity 187 member B	A0A8I3N5J1	-		X	
FAST kinase domains 5	A0A8I3NZU0	FASTKD5		X	
Fetuin B	A0A8I3P629	FETUB		X	

FHA domain-containing protein	A0A8I3PZW2	MKI67	X		
Fibronectin	A0A8I3PED8	FN1	X		
Fibrinogen C-terminal domain-containing protein	A0A8I3NLF4	FCN2			X
F-box and leucine rich repeat protein 8	A0A8I3NGN3	FBXL8			X
Focadhesin	A0A8I3NLD5	FOCAD			X
Globin A2	A0A1K0GGH0	GLNA2	X	X	X
Globin family profile domain-containing protein	A0A8I3MJ33	HBQ1			X
Glutamate receptor	A0A8I3MAF2	GRIA4	X		X
Glycerol-3-phosphate acyltransferase 1, mitochondrial	A0A8I3Q0U3	GPAM	X	X	X
Hemoglobin subunit alpha1	A0A5B8JID5	HBQ1	X	X	
Hyaluronoglucosaminidase	A0A8I3MPD2	CEMIP	X		
60S ribosomal protein L30	A0A8I3MP22	-		X	
IF rod domain-containing protein	A0A8I3PEZ2	-		X	
Ig heavy chain V region MOO	P01785	-	X		
Ig heavy chain V region GOM	P01784	-			X
Ig-like domain-containing protein	A0A8I3P6S7, A0A8I3PB96, A0A8I3P3K1, A0A8I3RX63, A0A8I3PVB5, A0A8I3S0K3, A0A8I3NU27, A0A8I3NRC4, A0A8I3NVL7	-	X	X	X
Immunoglobulin V-set domain-containing protein	A0A8I3PGC2	-			X
Joining chain of multimeric IgA and IgM	A0A8I3RW25	JCHAIN	X	X	X
Kelch domain containing 9	A0A8I3Q1J8	KLHDC9	X		
Keratin, type I cytoskeletal 9	A0A8I3NNW0	KRT9	X	X	X
Keratin, type I cytoskeletal 10	A0A8I3NL87	KRT10	X	X	X

Keratin, type II cytoskeletal 1	Q6EIY9	KRT1	X	X	X
Keratin, type II cytoskeletal 2 epidermal	Q6EIZ1	KRT2	X		
Keratin 18	A0A8I3PJ84	KRT18	X	X	X
Keratin 74	A0A8I3PMV1	KRT74	X	X	
Keratin 75	A0A8I3PK78	KRT6A	X	X	X
Kinesin-like protein	A0A8I3N413	KIF5B			X
Lysozyme	A0A8I3NX55	LYZ	X		
Leucyl and cystinyl aminopeptidase	A0A8I3ML59	LNPEP			X
Mitochondrial ribosomal protein L21	A0A8I3PYK8	LOC119868876			X
Mitochondrial ribosomal protein L43	A0A8I3P6K3	MRPL43	X		
Membrane bound O-acyltransferase domain containing 2	A0A8I3PNZ4	MBOAT2			X
Multifunctional ROCO family signaling regulator 1	A0A8I3NEN2	MFHAS1			X
NAD(P)(+)- arginine ADP-ribosyltransferase	A0A8I3Q484	ART3	X	X	
Nestin	A0A8I3N5W6	NES		X	
Ninein like	A0A8I3Q2Q5	NINL			X
NLR family pyrin domain containing 14	A0A8I3N8P2	NLRP14		X	
Nuclear receptor corepressor 2	A0A8I3NQ55	NCOR2			X
Peptidase S1 domain-containing protein	A0A8I3P0A5	LOC475521	X	X	X
Peptidase S1 domain-containing protein	A0A8I3MX03	LOC479668		X	X
Protocadherin 17	A0A8I3PDN1	PCDH17	X	X	X
Protocadherin 9	A0A8I3PB28	PCDH9		X	
POU domain protein	A0A8I3P3I7	LOC481709	X		
RAB23, member RAS onco family	A0A8I3NF40	RAB23		X	
receptor protein-tyrosine kinase	A0A8I3MB23	EPHA8	X	X	X
Ring finger protein 214	A0A8I3MLN4	R14			X
RING-type E3 ubiquitin transferase	A0A8I3N3Q3	TRIM17	X		

RUN and FYVE domain containing 1	A0A8I3N3X3	RUFY1	X	X	X
Secernin 2	A0A8I3NUU3	SCRN2			X
Serine/threonine-protein kinase greatwall	A0A8I3MPU6	MASTL			X
SET binding factor 2	A0A8I3NHN5	SBF2		X	
Solute carrier family 2 member 6	A0A8I3N592	SLC2A6	X		
SRC kinase signaling inhibitor 1	A0A8I3NHM6	SRCIN1		X	
Spectrin repeat containing nuclear envelope protein 2	A0A8I3MJS4	SYNE2			X
Sushi domain-containing protein	A0A8I3ML63	LOC100855476	X	X	X
Synaptic Ras GTPase activating protein 1	A0A8I3PL77	SYNGAP1		X	
Titin	A0A8I3PE72	TTN	X		
T-complex protein 1 subunit theta	A0A8I3P949	CCT8			X
Thymocyte selection associated high mobility group box	A0A8I3PZX4	TOX	X		
Transmembrane protein 191C	A0A8I3PRH2	TMEM191C			X
SEC63 homolog, protein translocation regulator	A0A8I3N658	SEC63	X		
Tubulin polymerization promoting protein family member 3	A0A8I3N563	TPPP3			X
Tumor protein p53 binding protein 1	A0A8I3S3V8	TP53BP1			X
Vang-like protein	A0A8I3Q337	VANGL1	X		
Vesicle amine transport 1	A0A8I3RT77	VAT1			X
WD repeat domain 41	A0A8I3N5E9	WDR41	X		
Uncharacterized protein	A0A5F4DJ18	-	X	X	X
Uncharacterized protein	A0A5F4BYG2	-			X

Table S4.
Gene ontology of proteins extracted from serum extracellular vesicles in dogs. Data obtained of UniprotKB (www.uniprot.org).

Protein Name	ID	Gene	Gene Ontology		
			**Molecular Function**	**Biological Process**	**Cellular Component**
Acetyl-CoA acyltransferase 2	A0A8I3NU61	ACAA2	Acyltransferase activity, transferring groups other than amino-acyl groups	-	-
A-kinase anchoring protein 3	A0A8I3Q3V5	AKAP3	Protein kinase A binding	Blastocyst hatching; protein localization transmembrane receptor protein serine/threonine kinase signaling pathway	Acrosomal vesicle; sperm fibrous sheath; sperm midpiece; sperm principal piece
Albumin	A0A8I3MY51	AFP	-	-	Extracellular space
Apolipoprotein E	P18649	APOE	Amyloid-beta binding; antioxidant activity; cholesterol transfer activity; enzyme binding; heparan sulfate proteoglycan binding; heparin binding; identical protein binding; lipoprotein particle binding; lipoprotein particle binding; lipoprotein particle binding; phosphatidylcholine-sterol O-acyltransferase activator activity; phospholipid binding; protein homodimerization activity; tau protein binding; very-low-density lipoprotein	AMPA glutamate receptor clustering; amyloid precursor protein metabolic process; artery morphogenesis; cGMP-mediated signaling; cholesterol catabolic process; cholesterol efflux; cholesterol homeostasis; chylomicron remnant clearance; fatty acid homeostasis; fatty acid homeostasis; gene expression; high-density lipoprotein particle assembly; high-density lipoprotein particle clearance; high-density lipoprotein particle remodeling; intermediate-density lipoprotein particle clearance intracellular calcium ion homeostasis; lipid transport involved in lipid storage; lipoprotein biosynthetic process; lipoprotein biosynthetic process; locomotory exploration behavior; long-chain fatty acid transport; long-term memory; long-term memory; maintenance of location in cell; melanosome organization; negative regulation	Chylomicron; extracellular exosome; extracellular space; Golgi apparatus; intermediate-density lipoprotein particle; low-density lipoprotein particle; melanosome; multivesicular body, internal vesicle; plasma membrane; synaptic cleft; very-low-density lipoprotein particle

				of amyloid fibril formation; negative regulation of amyloid-beta formation; negative regulation of blood vessel endothelial cell migration; negative regulation of canonical Wnt signaling pathway; negative regulation of cholesterol biosynthetic process; negative regulation of endothelial cell proliferation; negative regulation of gene expression; negative regulation of inflammatory response; negative regulation of inflammatory response; negative regulation of inflammatory response; negative regulation of neuron apoptotic process; negative regulation of neuron projection development; negative regulation of platelet activation; negative regulation of protein secretion; negative regulation of smooth muscle cell proliferation; negative regulation of triglyceride metabolic process; neuron projection development; nitric oxide mediated signal transduction; NMDA glutamate receptor clustering; phospholipid efflux; positive regulation by host of viral process; positive regulation by host of viral process; positive regulation of cholesterol efflux; positive regulation of cholesterol metabolic process; positive regulation of CoA-transferase activity; positive regulation of dendritic spine development; positive regulation of dendritic spine maintenance; positive regulation of DNA-templated transcription; positive regulation of endocytosis; positive regulation of endocytosis; positive regulation of heparan	

				sulfate binding; positive regulation of heparan sulfate proteoglycan binding; positive regulation of heparan sulfate proteoglycan binding; positive regulation of lipid transport across blood-brain barrier; positive regulation of low-density lipoprotein particle receptor catabolic process; positive regulation of membrane protein ectodomain proteolysis; positive regulation of neuron projection development; positive regulation of nitric-oxide synthase activity; positive regulation of phospholipid efflux; protein import receptor-mediated endocytosis; regulation of amyloid-beta clearance; regulation of behavioral fear response; regulation of Cdc42 protein signal transduction; regulation of cellular response to very-low-density lipoprotein particle stimulus; regulation of innate immune response; regulation of proteasomal protein catabolic process; regulation of protein-containing complex assembly; response to caloric restriction; response to dietary excess; response to dietary excess; reverse cholesterol transport; reverse cholesterol transport; triglyceride metabolic process; triglyceride-rich lipoprotein particle clearance; vasodilation; very-low-density lipoprotein particle clearance; very-low-density lipoprotein particle clearance virion assembly.	
Asparagine--tRNA ligase	A0A8I3MX22	NARS2	Asparagine-tRNA ligase activity; ATP binding; nucleic acid binding ;	Asparaginyl-tRNA aminoacylation	-

Beta 3-glucosyltransferase	A0A8I3PAR3	B3GLCT	-	Glycosyltransferase activity	Membrane
Cardiotrophin like cytokine factor 1	A0A8I3N6Q7	CLCF1	-	-	Extracellular region
Cupin superfamily member 1	A0A8I3MM27	C1H18orf54	-	-	-
C2 domain containing 3 centriole elongation regulator	A0A8I3MYR6	C2CD3	-	Brain development; centriole elongation; embryonic digit morphogenesis; heart looping; in utero embryonic development; neural plate axis specification; neural tube development; non-motile cilium assembly; protein localization to centrosome; protein processing; regulation of proteolysis; regulation of smoothened signaling pathway	Centriolar satellite; centriole; ciliary basal body
CD5 molecule like	A0A8I3NQW4	CD5L	Scavenger receptor activity	-	Membrane
Clock circadian regulator	A0A8I3NBB7		Cellular response to ionizing radiation; circadian regulation of gene expression; DNA damage checkpoint signaling; negative regulation of DNA-templated transcription; negative regulation of glucocorticoid receptor signaling pathway; positive regulation of circadian rhythm; positive regulation of inflammatory response; positive regulation of	Chromatin DNA binding; DNA-binding transcription activator activity, RNA polymerase II-specific; E-box binding; histone acetyltransferase activity; protein dimerization activity	Chromatoid body; chromosome; CLOCK-BMAL transcription complex; cytosol; nucleoplasm

			NF-kappaB transcription factor activity; proteasome-mediated ubiquitin-dependent protein catabolic process; protein acetylation; regulation of hair cycle; regulation of insulin secretion; regulation of type B pancreatic cell development; response to redox state; spermatogenesis		
C-type lectin domain-containing protein	A0A8I3PY16	CLEC1B	-	Receptor-mediated endocytosis	Membrane
DNA-(apurinic or apyrimidinic site) endonuclease	A0A8I3S7J9	APEX2	DNA binding; endonuclease activity;	DNA recombination; DNA repair	Fibrillar center; mitochondrion; nucleoplasm
DNA-directed RNA polymerase subunit beta	A0A8I3NPE6	POLR2B	Chromatin binding; DNA binding; DNA-directed 5'-3' RNA polymerase activity; metal ion binding; ribonucleoside binding	Transcription by RNA polymerase II	Chromosome, telomeric region; RNA polymerase II, core complex
Double-strand break repair protein	A0A8I3P7Y4	MRE11	3'-5'-DNA exonuclease activity; 5'-3' exonuclease activity; DNA binding; DNA helicase activity; identical protein binding; manganese ion binding; single-stranded DNA endodeoxyribonuclease activity	Cell population proliferation; DNA strand resection involved in replication fork processing; double-strand break repair via homologous recombination; double-strand break repair via nonhomologous end joining; homologous chromosome pairing at meiosis ; mitotic G2 DNA damage checkpoint signaling; mitotic intra-S DNA damage checkpoint signaling; negative regulation of	BRCA1-C complex; chromosome, telomeric region; cytoplasm; Mre11 complex; PML body; replication fork; site of double-strand break

				apoptotic process; positive regulation of kinase activity; positive regulation of protein autophosphorylation; positive regulation of telomere maintenance; sister chromatid cohesion; telomeric 3' overhang formation	
Dynamin 1 like	A0A8I3P3R6	Dynamin 1 like	GTP binding; GTPase activity	-	-
Dynein axonemal heavy chain 2	A0A8I3PUB4	Dynein axonemal heavy chain 2	ATP binding; dynein intermediate chain binding; dynein light intermediate chain binding; minus-end-directed microtubule motor activity	Cilium-dependent cell motility; inner dynein arm assembly; microtubule-based movement	Inner dynein arm; microtubule; sperm flagellum
Dystonin	A0A8I3NP68	DST	Actin binding; calcium ion binding; microtubule binding	Intermediate filament cytoskeleton organization	Cell projection; cytoplasm; membrane; microtubule
E3 ubiquitin-protein ligase	A0A8I3NIE2	DTX3	Metal ion binding; ubiquitin protein ligase activity	Notch signaling pathway; protein ubiquitination	Cytoplasm
EvC ciliary complex subunit 2	A0A8I3MIA3	EVC2	-	Smoothened signaling pathway	Cytoskeleton; cytoplasm; ciliary membrane
Family with sequence similarity 187 member B	A0A8I3N5J1	-	-	-	Membrane-
FAST kinase domains 5	A0A8I3NZU0	FASTKD5	Metal ion binding; ubiquitin-protein transferase activity	Protein ubiquitination	-
Fetuin B	A0A8I3P629	FETUB	Cysteine-type endopeptidase inhibitor activity	-	-
FHA domain-containing protein	A0A8I3PZW2	MKI67	-	Cell cycle	Nucleus

Fibronectin	A0A8I3PED8	FN1	Heparin binding; identical protein binding; integrin binding; peptidase activator activity; protease binding; proteoglycan binding	Acute-phase response; biological process involved in interaction with symbiont; calcium-independent cell-matrix adhesion; cell-substrate junction assembly; endodermal cell differentiation; integrin activation; integrin-mediated signaling pathway; negative regulation of monocyte activation; negative regulation of transforming growth factor beta production; neural crest cell migration involved in autonomic nervous system development; peptide cross-linking; positive regulation of axon extension; positive regulation of fibroblast proliferation; positive regulation of gene expression; positive regulation of phosphatidylinositol 3-kinase signaling; positive regulation of substrate-dependent cell migration, cell attachment to substrate; regulation of cell shape; regulation of ERK1 and ERK2 cascade; regulation of protein phosphorylation; substrate adhesion-dependent cell spreading; wound healing	Apical plasma membrane; basement membrane; endoplasmic reticulum-Golgi intermediate compartment; extracellular exosome; fibrinogen complex;
Fibrinogen C-terminal domain-containing protein	A0A8I3NLF4	FCN2	-	-	-
F-box and leucine rich repeat protein 8	A0A8I3NGN3	FBXL8	-	-	-
Focadhesin	A0A8I3NLD5	FOCAD	-	Regulation of post-transcriptional gene silencing	-

Globin A2	A0A1K0GGH0	GLNA2	Heme binding; metal ion binding; oxygen binding; oxygen carrier activity	-	Hemoglobin complex
Globin family profile domain-containing protein	A0A8I3MJ33	HBQ1	Heme binding; iron ion binding; oxygen carrier activity; oxygen binding	-	Hemoglobin complex
Glutamate receptor	A0A8I3MAF2	GRIA4	Ionotropic glutamate receptor activity	-	Postsynaptic membrane
Glycerol-3-phosphate acyltransferase 1, mitochondrial	A0A8I3Q0U3	GPAM	Glycerol-3-phosphate O-acyltransferase activity	CDP-diacylglycerol biosynthetic process	Mitochondrial outer membrane; plasma membrane
Hemoglobin subunit alpha1	A0A5B8JID5	HBQ1	Heme binding; iron ion binding; oxygen carrier activity; oxygen binding	-	Hemoglobin complex
Hyaluronoglucosaminidase	A0A8I3MPD2	CEMIP	-	-	Plasma membrane
60S ribosomal protein L30	A0A8I3MP22	-	RNA binding; structural constituent of ribosome	-	Cytosolic large ribosomal subunit
IF rod domain-containing protein	A0A8I3PEZ2	-	-	-	Intermediate filament
Ig heavy chain V region MOO	P01785	-	-	Adaptive immune response	External side of plasma membrane; immunoglobulin complex

Ig heavy chain V region GOM	P01784	-	-	Adaptive immune response	External side of plasma membrane; immunoglobulin complex
Ig-like domain-containing protein	A0A8I3P6S7, A0A8I3PB96, A0A8I3P3K1, A0A8I3RX63, A0A8I3PVB5, A0A8I3S0K3, A0A8I3NU27, A0A8I3NRC4, A0A8I3NVL7	-	-	-	-
Immunoglobulin V-set domain-containing protein	A0A8I3PGC2	-	-	-	-
Joining chain of multimeric IgA and IgM	A0A8I3RW25	JCHAIN	IgA binding; immunoglobulin receptor binding; peptidoglycan binding; phosphatidylcholine binding; protein homodimerization activity; protein-macromolecule adaptor activity; single-stranded DNA binding;	Adaptive immune response; antibacterial humoral response; glomerular filtration; innate immune response; positive regulation of respiratory burst; protein-containing complex assembly	Monomeric IgA immunoglobulin complex; pentameric IgM immunoglobulin complex; secretory dimeric IgA immunoglobulin complex
Kelch domain containing 9	A0A8I3Q1J8	KLHDC9	-	-	-
Keratin, type I cytoskeletal 9	A0A8I3NNW0	KRT9	Structural molecule activity	Intermediate filament organization; skin development; spermatogenesis	Intermediate filament

Keratin, type I cytoskeletal 10	A0A8I3NL87	KRT10	Protein heterodimerization activity; structural constituent of skin epidermis	Keratinocyte differentiation; peptide cross-linking; protein heterotetramerization	Cornified envelope; cytoplasm; keratin filament
Keratin, type II cytoskeletal 1	Q6EIY9	KRT1	Protein heterodimerization activity; structural constituent of skin epidermis	Keratinization; protein heterotetramerization	Cytoplasm; keratin filament; plasma membrane
Keratin, type II cytoskeletal 2 epidermal	Q6EIZ1	KRT2	Structural constituent of skin epidermis	Structural constituent of skin epidermis; positive regulation of epidermis development	Cytoplasm; keratin filament
Keratin 18	A0A8I3PJ84	KRT18	Structural molecule activity	-	Intermediate filament
Keratin 74	A0A8I3PMV1	KRT74	Keratin filament binding	Intermediate filament cytoskeleton organization	Cytoplasm; keratin filament
Keratin 75	A0A8I3PK78	KRT6A	-	Hematopoietic progenitor cell differentiation	Cornified envelope; keratin filament
Kinesin-like protein	A0A8I3N413	KIF5B	ATP binding; identical protein binding; microtubule binding; microtubule motor activity; protein-containing complex binding;	Cellular response to type II interferon; centrosome localization; cytoplasm organization; lysosome localization; mitochondrion transport along microtubule; mitocytosis; natural killer cell mediated cytotoxicity; plus-end-directed vesicle transport along microtubule; positive regulation of potassium ion transport; positive regulation of protein localization to plasma membrane; positive regulation of synaptic transmission, GABAergic; positive regulation of synaptic transmission, GABAergic; stress granule disassembly	Centriolar satellite; ciliary rootlet; cytosol; microtubule; mitochondrion; neuron projection; phagocytic vesicle
Lysozyme	A0A8I3NX55	LYZ	Identical protein binding; lysozyme activity	Defense response to Gram-positive bacterium; killing of cells of another organism	Extracellular space

Leucyl and cystinyl aminopeptidase	A0A8I3ML59	LNPEP	Aminopeptidase activity; metallopeptidase activity; zinc ion binding	Proteolysis	Membrane
Mitochondrial ribosomal protein L21	A0A8I3PYK8	LOC119868876	-	-	Cytoplasm; ribosome
Mitochondrial ribosomal protein L43	A0A8I3P6K3	MRPL43	Structural constituent of ribosome	Mitochondrial translation	Mitochondrial large ribosomal subunit
Membrane bound O-acyltransferase domain containing 2	A0A8I3PNZ4	MBOAT2	-	-	Membrane
Multifunctional ROCO family signaling regulator 1	A0A8I3NEN2	MFHAS1	GTP binding; protein phosphatase 2A binding; ubiquitin protein ligase binding	Erythrocyte differentiation; inflammatory response; innate immune response; negative regulation of inflammatory response; negative regulation of protein dephosphorylation; negative regulation of protein localization to nucleus; negative regulation of toll-like receptor 2 signaling pathway; negative regulation of toll-like receptor 4 signaling pathway; positive regulation of ERK1 and ERK2 cascade; positive regulation of JNK cascade; positive regulation of p38MAPK cascade; positive regulation of protein kinase B signaling; positive regulation of toll-like receptor 2 signaling pathway; positive regulation of toll-like receptor 2 signaling pathway; regulation of toll-like receptor signaling pathway	Cytoplasm

NAD(P)(+)-arginine ADP-ribosyltransfe rase	A0A8I3Q484	ART3	NAD+-protein-arginine ADP-ribosyltransferase activity; nucleotidyltransferase activity	-	Membrane
Nestin	A0A8I3N5W6	NES	Intermediate filament binding	G2/M transition of mitotic cell cycle; negative regulation of catalytic activity; negative regulation of protein binding; stem cell proliferation	Cytoplasm; intermediate filament
Ninein like	A0A8I3Q2Q5	NINL	Calcium ion binding	-	Cytosol; intercellular bridge; microtubule cytoskeleton
NLR family pyrin domain containing 14	A0A8I3N8P2	NLRP14	-	Spermatogenesis	-
Nuclear receptor corepressor 2	A0A8I3NQ55	NCOR2	-	-	-
Peptidase S1 domain-containing protein	A0A8I3P0A5	LOC4755 21	Serine-type endopeptidase activity	Proteolysis	-
Peptidase S1 domain-containing protein	A0A8I3MX03	LOC4796 68	Hemoglobin binding; serine-type endopeptidase activity	Proteolysis	Extracellular region
Protocadheri n 17	A0A8I3PDN1	PCDH17	Calcium ion binding	Homophilic cell adhesion via plasma membrane adhesion molecules	Plasma membrane
Protocadheri n 9	A0A8I3PB28	PCDH9	Calcium ion binding	Homophilic cell adhesion via plasma membrane adhesion molecules	Cell-cell contact zone; growth cone; plasma membrane

POU domain protein	A0A8I3P3I7	LOC481709	DNA binding; DNA-binding transcription factor activity, RNA polymerase II-specific	-	Nucleus
RAB23, member RAS onco family	A0A8I3NF40	RAB23	GTP binding; GTPase activity	-	-
Receptor protein-tyrosine kinase	A0A8I3MB23	EPHA8	ATP binding; GPI-linked ephrin receptor activity; growth factor binding	Axon guidance; cellular response to follicle-stimulating hormone stimulus; neuron remodeling; positive regulation of MAPK cascade; positive regulation of phosphatidylinositol 3-kinase activity; protein autophosphorylation; regulation of cell adhesion mediated by integrin; substrate-dependent cell migration	Neuron projection; plasma membrane
Ring finger protein 214	A0A8I3MLN4	RNF214	Metal ion binding	-	-
RING-type E3 ubiquitin transferase	A0A8I3N3Q3	TRIM17	Zinc ion binding	-	Cytoplasm
RUN and FYVE domain containing 1	A0A8I3N3X3	RUFY1	Metal ion binding; SH2 domain binding; SH3 domain binding	Protein transport; regulation of endocytosis; small GTPase mediated signal transduction	Cytosol; endosome; nuclear speck
Secernin 2	A0A8I3NUU3	SCRN2	Cysteine-type exopeptidase activity; dipeptidase activity	Proteolysis	-
Serine/threonine-protein kinase greatwall	A0A8I3MPU6	MASTL	ATP binding; protein serine/threonine kinase activity	Female meiosis II; positive regulation of ubiquitin protein ligase activity; protein phosphorylation	Microtubule organizing center; nucleus
SET binding factor 2	A0A8I3NHN5	SBF2	Guanyl-nucleotide exchange factor activity	-	-

Solute carrier family 2 member 6	A0A8I3N592	SLC2A6	Transmembrane transporter activity	-	Membrane
SRC kinase signaling inhibitor 1	A0A8I3NHM6	SRCIN1	Protein kinase binding	Protein kinase binding; positive regulation of protein tyrosine kinase activity; postsynaptic actin cytoskeleton organization; regulation of cell migration; regulation of dendritic spine morphogenesis; substrate adhesion-dependent cell spreading	Actin cytoskeleton; cytoplasm; glutamatergic synapse
Spectrin repeat containing nuclear envelope protein 2	A0A8I3MJS4	SYNE2	Actin binding; cytoskeleton-nuclear membrane anchor activity	Centrosome localization; nuclear migration along microfilament; nucleokinesis involved in cell motility in cerebral cortex radial glia guided migration; positive regulation of cell migration; regulation of cilium assembly	Filopodium membrane; focal adhesion; intermediate filament cytoskeleton; lamellipodium membrane; meiotic nuclear membrane microtubule tethering complex; mitochondrion; nuclear lumen; nuclear membrane; sarcoplasmic reticulum; Z disc
Sushi domain-containing protein	A0A8I3ML63	LOC100855476	-	Involved in many recognition processes, including the binding of several complement factors to fragments C3b and C4b.	-
Synaptic Ras GTPase activating protein 1	A0A8I3PL77	SYNGAP1	GTPase activator activity;	GTPase activator activity; regulation of GTPase activity	-
Titin	A0A8I3PE72	TTN	ATP binding; protein kinase activity	Protein phosphorylation	Intracellular organelle

T-complex protein 1 subunit theta	A0A8I3P949	CCT8	ATP binding; ATP hydrolysis activity; ATP hydrolysis activity; unfolded protein binding	Binding of sperm to zona pellucida; chaperone-mediated protein folding; pore complex assembly; positive regulation of establishment of protein localization to telomere; positive regulation of telomere maintenance via telomerase; protein stabilization; toxin transport	Cell body; centrosome; chaperonin-containing T-complex; cilium; intermediate filament cytoskeleton; microtubule; microtubule; zona pellucida receptor complex
Thymocyte selection associated high mobility group box	A0A8I3PZX4	TOX	Chromatin DNA binding	CD4-positive, CD25-positive, alpha-beta regulatory T cell lineage commitment; CD8-positive, alpha-beta T cell lineage commitment; cerebral cortex neuron differentiation; lymph node development; natural killer cell differentiation; NK T cell lineage commitment; nucleic acid metabolic process; Peyer's patch development; positive regulation of DNA demethylation; positive regulation of natural killer cell differentiation; positive regulation of neural precursor cell proliferation; positive regulation of neuron projection development; regulation of positive thymic T cell selection	Nucleus
Transmembrane protein 191C	A0A8I3PRH2	TMEM191C	-		-
SEC63 homolog, protein translocation regulator	A0A8I3N658	SEC63	-	Liver development; liver development; multicellular organism aging; nitrogen compound metabolic process; post-translational protein targeting to membrane, translocation; SRP-dependent cotranslational protein targeting to membrane	Endoplasmic reticulum; membrane

Tubulin polymerization promoting protein family member 3	A0A8I3N563	TPPP3	Tubulin binding	Decidualization; embryo implantation; microtubule bundle formation; icrotubule polymerization	Microtubule bundle; perinuclear region of cytoplasm
Tumor protein p53 binding protein 1	A0A8I3S3V8	TP53BP1	-	DNA damage	-
Vang-like protein	A0A8I3Q337	VANGL1	-	Pigmentation	Lateral plasma membrane
Vesicle amine transport 1	A0A8I3RT77	VAT1	Oxidoreductase activity; zinc ion binding	Negative regulation of mitochondrial fusion	-
WD repeat domain 41	A0A8I3N5E9	WDR41	-	Regulation of autophagy	-
Uncharacterized protein	A0A5F4DJ18	-	-	Immune response	Extracellular space
Uncharacterized protein	A0A5F4BYG2	-	-	Immune response	Extracellular space
